# Bibliography

**Published:** 1891-10

**Authors:** 


### BIBLIOGRAPHY.

Therapeutics: Its Principles and Practice. By H. C. Wood, M.D., LL.D. The Eighth Edition of a Treatise on Therapeutics, Rearranged, Rewritten, and Enlarged. J. B. Lippincott Company. Philadelphia, 1891.

This standard work comes to us now in its eighth edition. It has long since passed beyond criticism, and is universally regarded as an authority on all subjects upon which it treats. The original work of the author has contributed much to advance therapeusis to something akin to scientific order, and to raise it from the condition he so forcibly describes in the preface to the first edition: "What to-day is believed is to-morrow to be cast aside. . . . . What has clinical therapeutics established permanently and indisputably? Scarcely anything beyond the primary facts that quinia will arrest an intermittent, that salts will purge, and that opium will quiet pain and lull to sleep.

"To established therapeutic facts the profession clings as with the heart and hand of one man,—clings with a desperation and unanimity whose intensity is the measure of the unsatisfied desire for something fixed. Yet with what a Babel of discordant voices does it celebrate its two thousand years of experience!"

Whether the time will come when the empiric use of drugs will give way to at least an intelligent conception of what is required remains a still unsolved problem; but the tendency to use other means is developing, and this is recognized by the author, who writes in his introduction, "In the treatment of chronic disease the best results are to be achieved by the regulation of the habits and mode of life of the patient, and by the employment of certain remedial measures other than drugs. Of late years the importance of modes of relief other than medication has so grown in my estimation that I have concluded in this edition to consider them at some length."

This edition has not been subjected to the "revolutionary changes" of the seventh, but there has been added the following subjects, re-written and "practically new:" "The whole subject of anæsthetics, the articles upon cocaine, strophanthus, caffeine, antipyrin, antifebrin, phenacetin, hydrastine, paraldehyd, lead-poisoning, etc. Among the absolutely new articles may be mentioned sulphonal, chloralamid, aristol, and others."

Objection may be made to the narrow limits to which the author confines the word therapeusis, and the relegation of disinfectants and antiseptics to hygiene. "It is evident," he writes, "that the consideration of these materials belongs to the province of hygiene rather than to that of therapeutics, since their employment is hygienic rather than medicinal, preventive rather than curative."

While this view may have been true in the past, it is not wholly so at the present, and the therapeusis of the future must concern itself with the removal of causes of disease if it is ever to accomplish anything upon a scientific basis. It may be difficult to destroy the pathogenic forms that originate many of the diseases, but it does seem unwise to assume that treatment here is "preventive rather than curative." The clinical observations in local treatment have demonstrated beyond cavil that the destruction of pathogenic bacteria in diseased tissues means a rapid restoration to normal condition, and if true here it may be made true in the broader sense in general treatment.

With this pronounced view of the author, this chapter will not be found to be entirely satisfactory. That portion devoted to peroxide of hydrogen is meagre in details. In view of the extended application of this agent, and its recognized value in the treatment of some forms of disease, a fuller description would have been valuable.

The use of tincture of chloride of iron is described as being "very destructive to the teeth; care ought to be exercised in its use about the mouth, and also in its administration." As dentists, we have reason to complain of this slight allusion to one of the most destructive agents used in the treatment of disease. There is no one that has given dentists more trouble, or that has been used by the medical profession with more indifference. It is certainly time that works of this character should teach that so injurious is the tincture of chloride of iron to the teeth, that some other form of iron should be substituted; or, if this be not possible, minute description of modes to combat its injurious effects should be given, as the use of antacids. We would refer the author to the pages of the August and September numbers of the JOURNAL for some forcible facts in this connection.

While there may still be "Babel" in therapeutics, there is so much of order in this work that it lifts the treatment of disease nearer to scientific methods, and gives promise of the possibilities of the future.

It scarcely need be said of anything to which Professor Wood places his name that it should be in the hands of all practitioners, whether in general or special practice. They cannot be without this work and be intelligent upon the subjects on which it treats.

